# Viromes of Monocotyledonous Weeds Growing in Crop Fields Reveal Infection by Several Viruses Suggesting Their Virus Reservoir Role

**DOI:** 10.3390/plants13182664

**Published:** 2024-09-23

**Authors:** Zsuzsanna N. Galbács, Evans Duah Agyemang, György Pásztor, András Péter Takács, Éva Várallyay

**Affiliations:** 1 Genomics Research Group, Department of Plant Pathology, Institute of Plant Protection, Hungarian University of Agriculture and Life Sciences, Szent-Györgyi Albert Street 4, H-2100 Godollo, Hungary; nagyne.galbacs.zsuzsanna@uni-mate.hu; 2 Department of Plant Protection, Institute of Plant Protection, Hungarian University of Agriculture and Life Sciences, H-8360 Keszthely, Hungary; evansagyemang932@gmail.com (E.D.A.); pasztor.gyorgy@uni-mate.hu (G.P.); takacs.andras.peter@uni-mate.hu (A.P.T.)

**Keywords:** metagenome, survey, virus, plant, weed, reservoir, HTS

## Abstract

In 2019, random samples of *Panicum miliaceum* growing as a weed were surveyed to uncover their virus infections at two locations in Hungary. This pilot study revealed infection with three viruses, two appearing for the first time in the country. As follow-up research, in the summer of 2021, we collected symptomatic leaves of several monocotyledonous plants in the same locations and determined their viromes using small RNA high-throughput sequencing (HTS). As a result, we have identified the presence of wheat streak mosaic virus (WSMV), barley yellow striate mosaic virus (BYSMV), barley virus G (BVG), and two additional viruses, namely Aphis glycines virus 1 (ApGlV1) and Ljubljana dicistrovirus 1 (LDV1), which are described for the first time in Hungary. New hosts of the viruses were identified: *Cynodon dactylon* is a new host of BYSMV and LDV1, *Echinocloa crus-galli* is a new host of BVG, ApGlV1 and LDV1, *Sorghum halepense* is a new host of ApGlV1, and *Panicum miliaceum* is a new host of LDV1. At the same time, *Zea mays* is a new host of ApGlV1 and LDV1. Small RNA HTS diagnosed acute infections but failed to detect persistent ones, which could be revealed using RT-PCR. The infection rates at the different locations and plant species were different. The phylogenetic analyses of the sequenced virus variants suggest that the tested monocotyledonous weeds can host different viruses and play a virus reservoir role. Viral spread from the reservoir species relies on the activity of insect vectors, which is why their management requires an active role in plant protection strategies, which need careful planning in the changing environment.

## 1. Introduction

Several biotic factors threaten the efficient cultivation of cereals. Weeds growing together with crops can compete for food and water supply, affecting crops’ growth and development. Virus infection can disturb the physiology of the plants and could lead to the development of various symptoms. Altered growth and symptoms could lead to deteriorated and decreased yield and result in economic loss. 

Weeds can be infected with viruses and can serve as a shelter and food for the virus vector insects. In contrast to the annual crops, they can be perennial and overwinter in the fields, occasionally in virus-infected forms. These plants constitute potential reservoirs for viruses that may spread into cultivated crops, thereby leading to epidemics or the emergence of novel viruses [1]. Most plant viruses are transmitted by insect vectors, which may increase the possibilities for virus transmission across landscapes and the distances over which viruses can be transmitted. Seed transmission is usually rare, depends upon the virus species, and is influenced by several abiotic parameters. Climatic variations can contribute to the successful spread of newly introduced viruses or their vectors, as can the presence of these organisms in new environments that were previously unfavourable for them. 

Plant viruses can spill over in both directions between weed plants and crops with potential adverse effects in both managed and natural ecosystems. The spillover of viruses onto cultivated plants usually occurs at the borders of plant ecosystems [2,3]. However, “introduced” viruses can adapt to further transmissions in a new host, leading to emerging viral infections [4]. While herbicides can regulate weeds, there is no chemical protection against viral infections. In these cases, protection against insect vectors is the generally accepted strategy.

Plant virus ecology, investigating plants without any hint of virus infection, is a recent field of study [5,6]. The emergence of high-throughput sequencing (HTS) methods offering optimal diagnostics to describe the viromes of plants growing in natural habitats [7] makes it possible to investigate this question in detail [8]. Weeds growing near crop fields can host viruses in unexpected numbers [9].

The virus reservoir role of grasses, growing in and at the edge of cereal fields, has been under investigation for a long time, and their potential role as virus reservoirs in the Baltic states [10], the UK [11], and Sweden has been revealed [12].

During a proof-of-concept pilot study, the viromes of *Panicum miliaceum* (millet), grown as weeds were determined by small RNA (sRNA) HTS [13]. As a result, from the pools of 20 millet samples randomly collected at two locations, we identified the presence of the wheat streak mosaic virus (WSMV), the barley yellow striate mosaic virus (BYSMV), and the barley virus G (BVG), with the latter two appearing for the first time in Hungary. 

WSMV, a typical potyvirus, a member of the *Tritimovirus* genus, has a linear positive sense, 9339–9383 nt long RNA genome, encoding one large polyprotein [14]. It originates from Asia and was introduced to America as germplasm from the Black Sea region in the last century, possibly with its vector [15]. It is not considered an invasive pest; however, it can be transmitted through the seeds at a low rate [16], which could be an important inoculum source when the wheat curl mite (WCM—*Aceria tosichella*), its main polyphagous vector [17], is present [18]. WSMV is a devastating pathogen in wheat-growing regions globally and was diagnosed in Hungary in the 1980s [19]. The host range of the virus and its vector is wide, including millet and several different grass species, even perennial ones, which can overwinter [14,20]. In the NCBI GenBank, WSMV sequences from *Alopecurus pratensis*, *Agropyron repens*, *Avena fatua*, *Echinochloa crus-galli*, *Lolium* sp., *Phleum pratense*, *Phragmites australis*, and *Poa pratensis* from different countries of origin are deposited (see Table S1 for their accession numbers and citations).

BYSMV, a *Cytorhabdovirus*, possessing a negative single-stranded RNA genome, was sequenced in 2015 [21] but was described a long time ago in Italy and France with its planthopper vector (*Laodelphax striatellus*) [22]. BYSMV has been identified in Morocco [23], Iran [24], Turkey [25], Syria [26], China [27], and, during our pilot study, in Hungary [13]. Besides its main cereal hosts of wheat and barley, it can infect millet [13,24] and *Setaria italica* [28] (Table S1). Maize sterile stunt virus (MSSV) infecting *Zea mays* (maize) was present in Australia and turned out to be a distant strain of BYSMV, suggesting the wider diversity of the virus [29].

BVG is a *Polerovirus* (family *Luteoviridae*), with a positive sense RNA genome, encoding six open reading frames, identified in barley in South Korea using HTS [30], where it has also been shown to infect *P. miliaceum* [31] and *S. italica* [32]. It has been found to infect barley and oats in Australia [33] and barley in the US [34] and has been reported in symptomatic switchgrass imported from the Netherlands [35]. It is present in Europe, as BVG has been reported in millet in Hungary [13], in winter barley in France [36], and in weed species in Germany (Gaafar and Ziebell, 2019, only GenBank record) and Slovenia [9] (Table S1). BVG has been detected and sequenced in *Rhopalosiphum maidis* and *Rhopalosiphum padi* (South Korea—Jeong and Lee, 2021, only GenBank Record), suggesting its possible virus vector role, which was proven using an infectious cDNA clone of BVG [37].

ApGlV1 is a *Dicipivirus* (order *Picornavirales*), with a sense RNA genome encoding two polyproteins, and was first found in the US using HTS in parallel in *Glycine max* and *Aphis glycines* [38]. It has been found in a laboratory *T. urticae* population, established from insect populations originating from Spain, the US, and Japan [39]. Moreover, ApGlV1 has been found in Slovenia in tomato and mixed weed samples containing the RNA of eight species [9] (Table S1).

LDV1 is a *Dicistrovirus* (order *Picornavirales*), possessing a sense RNA genome and encoding an RdRp and a coat protein. It has been identified in Slovenia in pooled weed samples, containing the RNA of seven plant species [9] (Table S1).

Based on the result of our pilot study, we suspected that WSMV, BYSMV, and BVG could have overwintered in millet or other monocotyledonous weeds growing in these fields. This research aims to determine the viromes of monocotyledonous weeds present in the same locations using sRNA HTS and RT-PCR. In the investigated six plant species, the presence of five viruses was identified, representing not only the first description of ApGlV1 and LDV1 in Hungary but also identifying new hosts for most of the viruses.

## 2. Results

### 2.1. Small RNA HTS of the Weeds Revealed the Presence of Five Viruses

During our pilot study, a survey carried out in 2019 [13], we found WSMV, BYSMV, and BVG in millet growing as a weed. As a follow-up study in July of 2021, we collected monocotyledonous weeds at the same two locations, as well as at an additional location, to check their viromes using sRNA HTS (Figure S1). Besides sampling millet and cultivated maize, the symptomatic leaves of weeds growing at US and BA, namely *Echinocloa crus-galli*, *Setaria viridis*, *Cynodon dactylon*, and *Sorghum halepense*, were sampled (Figure S2). 

**Figure 1:**
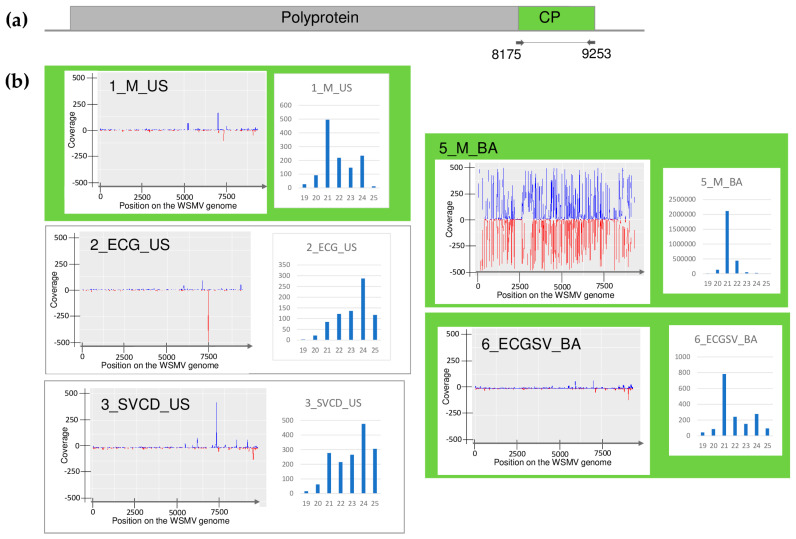
Investigation of the mapped viral reads in the case of WSMV. (**a**) shows the genome organization, while (**b**) shows the coverage of the viral genome by sRNAs and the size distribution of the viral reads in the indicated libraries. Plots are presented for libraries where the presence of the virus has been detected by RT-PCR. Green marks libraries where sRNA HTS detected the presence of the virus. On (**a**) CP encodes the coat protein of the virus. On (**b**) blue marks the small RNAs with sense and red with antisense orientation.

**Figure 2:**
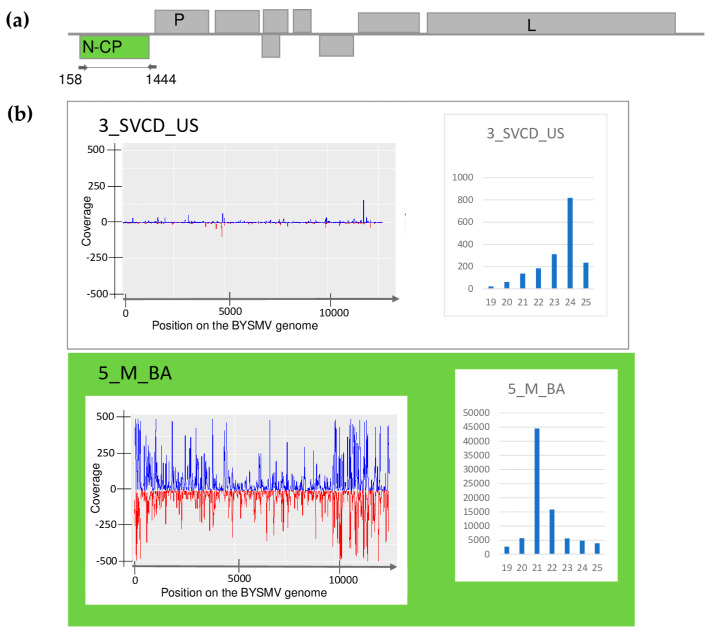
Investigation of the mapped viral reads in the case of BYSMV (**a**) shows the genome organization, while (**b**) shows the coverage of the viral genome by sRNAs and the size distribution of the viral reads in the indicated libraries. Plots are presented for libraries where the presence of the virus has been detected by RT-PCR. Green marks libraries where sRNA HTS detected the presence of the virus. On (**a**) P and L encode the P and L protein and N-CP is the coat protein of the virus. On (**b**) blue marks the small RNAs with sense and red with antisense orientation.

**Figure 3:**
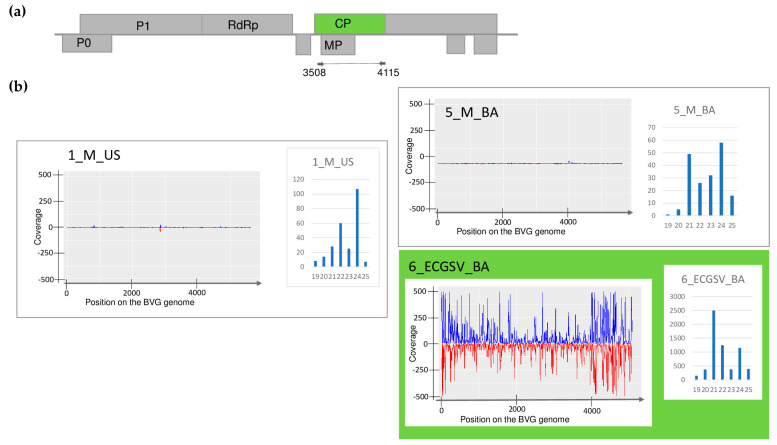
Investigation of the mapped viral reads in the case of BVG (**a**) shows the genome organization, while (**b**) shows the coverage of the viral genome by sRNAs and the size distribution of the viral reads in the viral reads in the indicated libraries. Plots are presented for libraries where the presence of the virus has been detected by RT-PCR. Green marks libraries where sRNA HTS detected the presence of the virus. On (**a**) P0 and P1 encodes the P0 and P1 protein, RdRP is the RNA-dependent RNA polymerase, MP is the movement protein, and CP is the coat protein of the virus. On (**b**) blue marks the small RNAs with sense and red with antisense orientation.

**Figure 4:**
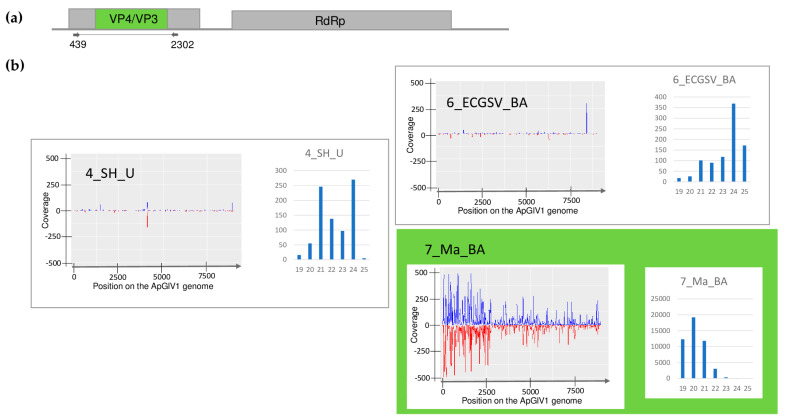
The investigation of the mapped viral reads in the case of ApGlV1 (**a**) shows the genome organization, while (**b**) shows the coverage of the viral genome by sRNAs and the size distribution of the viral reads in the indicated libraries. Plots are presented for libraries where the presence of the virus has been detected by RT-PCR. Green marks the libraries where sRNA HTS detected the presence of the virus. On (**a**) VP4 and VP3 encodes the P4 and P3 protein, and RdRP is the RNA-dependent RNA polymerase of the virus. On (**b**) blue marks the small RNAs with sense and red with antisense orientation.

**Figure 5:**
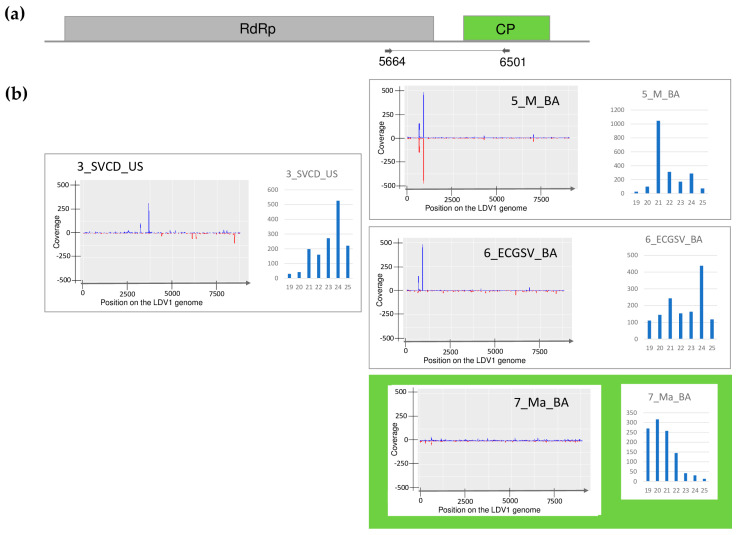
The investigation of the mapped viral reads in the case of LDV1 (**a**) shows the genome organization, while (**b**) shows the coverage of the viral genome by sRNAs and the size distribution of the viral reads in the indicated libraries. Plots are presented for libraries where the presence of the virus has been detected by RT-PCR. Green marks libraries where sRNA HTS detected the presence of the virus. On (**a**) RdRP encodes the RNA-dependent RNA polymerase, and CP is the coat protein of the virus. On (**b**) blue marks the small RNAs with sense and red with antisense orientation.

The plants showed different symptoms including mosaic, leaf deformation, chlorosis, necrosis, and stunting, as shown in Figure S2a–i and detailed in Table A1. To reveal the viromes of the sampled plants, sRNA HTS was used as an unbiased method. Seven libraries, representing the sampled species according to their sampling area were sequenced using the Illumina Platform (see Table A1 for the library names and the pooling strategy). After the trimming and quality control of the sequenced 18–38 million reads, 17–37 million redundant reads remained, representing 1.5–4.2 million small RNA sequences (Table S2). From these non-redundant reads, 3103–20,797 contigs could be assembled, and using BLAST, were compared to the reference genomes of known land plant-infecting viruses. In addition, we searched for infection with recently described viruses using the virus database containing the full genomes of 165 viral strains described from Slovenian rural samples [9], hypothesising that the viromes of rural samples in Slovenia and Hungary, two neighbouring countries, could share some similarities. As a result, we obtained contig hits with acceptable *E*-values (lower than 10^−5^) for five viruses: WSMV (in 5_M_BA), BYSMV (5_M_BA), BVG (in 6_ECGSV_BA), ApGlV1, and LDV1 (both in the same library: 7_Ma_BA) (Table S3). The investigation of the distribution of the mapped viral reads to the viral genome revealed that the viral genome was highly covered (coverage was higher than 90% in the cases of WSMV, BYSMV, BVG, and ApGlV1, and was higher than 70% in the case of LDV1) (Figure 1, Figure 2, Figure 3, Figure 4 and Figure 5). While in these libraries, the number of the normalised redundant reads reached our set limit in the cases of WSMV (128,204), BYSMV (4446), BVG (212), and ApGlV1 (2359), in the case of LDV1, it was below this limit (87).

The size distribution of the viral mapped reads in these libraries, in the case of WSMV, BYSMV, and BVG showed that most of them were 21 nt long (Figure 1, Figure 2 and Figure 3, indicated by green), while in the case of ApGlV1 and LDV1, most of the reads were 19–21 nt long and the majority of these were 20 nt long (Figure 4 and Figure 5, indicated by green).

### 2.2. Validation of the sRNA HTS Using RT-PCR Revealed the Infection of the Five Investigated Viruses in More Hosts

**Figure 6:**
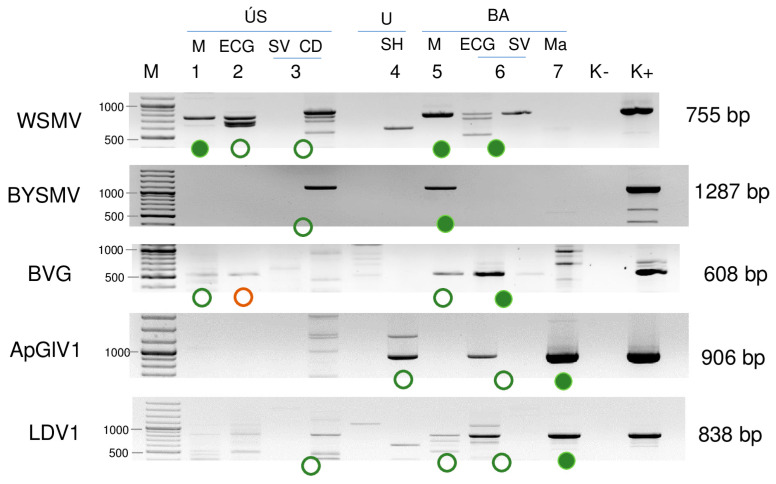
Virus diagnostics using RT-PCR and virus-specific primers. Circles indicate positive results, green when Sanger sequencing confirmed the presence of the virus, and red when Sanger sequencing did not confirm the RT-PCR result. Each library is represented only by one circle. Filled circles show cases with the same sRNA HTS result. M—stands for a GenRuler 100 bpPlus, used as a molecular marker. ÚS—Újmajor susnyás; U—Újmajor; BA—Büdös árok; M—*P. miliaceum*; ECG—*E. crus-galli*; SV—*S. viridis*; CD—*C. dactylon*; Ma—*Z. mays*. K− is the negative control, while K+ is the positive control.

To validate the result of the sRNA HTS, an independent method, RT-PCR was used. For the amplification, the primers of our previous study (in the cases of WSMV, BYSMV, and BVG) or newly designed ones (in the cases of ApGlV1 and LDV1) were used. To avoid the possibility of mis- and failed priming, because of the presence of different strains with slight differences, during the primer design, the sequences of the contigs and consensus sequences originating from the HTS were considered (Table S4). The result of the virus-specific RT-PCR showed a different viral landscape than sRNA HTS (Figure 6). 

The results of the sRNA HTS were successfully validated. Moreover, all of the viruses were detected in additional libraries: WSMV in 2_ECG_US and 3_SVCD_US; BYSMV in 3_SVCD_US; BVG in 1_M_US; 2_ECG_US; 5_M_BA; ApGlV1 in 4_SH_U and 6_ECGCV_BA; and LDV1 in 3_SVCD_US, 5_M_BA, and 6_ECGCV_BA.

To test how many individuals are infected with a particular virus in the pools, they were tested using RT-PCR. To achieve a simpler presentation, we will discuss this part of the results in terms of the viruses.

#### 2.2.1. WSMV Is Present at Both Locations in Millet and Also in Several Weed Species

**Figure 7:**
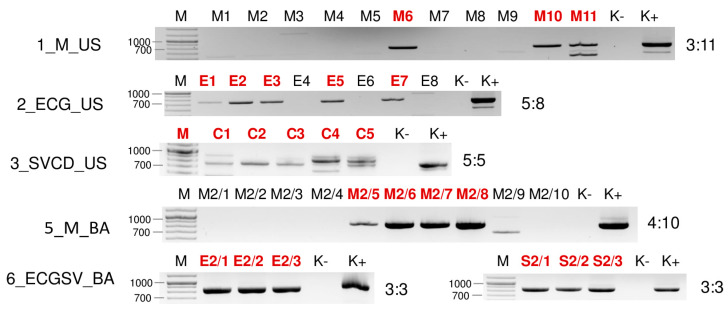
Virus diagnostics using RT-PCR to test the presence of WSMV in the sampled individuals amplifying a 755 bp long part of the WSMV genome using WSMV_8499F and WSMV_9253R primers (Sequences are available in Table S4). M—stands for a GenRuler 100 bpPlus, used as a molecular marker. K− is the negative, while K+ is the positive control. Red indicates positive individuals. The codes of the sRNA HTS libraries and the numbered individuals are marked based on Table A1. The infection rate of the plant species is also indicated.

Testing the presence of WSMV in the species pools using RT-PCR revealed the infection of millet and *E. crus-galli* at both locations: *C. dactylon* in US and *S. viridis* in BA (Figure 6). While all of the tested *C. dactylon* plants in US and all of the *E. crus-galli* and *S. viridis* plants in BA were infected, the infection rate was not 100% in the other cases: three millet (out of eleven) and five *E. crus-galli* samples in US (out of 8) and four millet samples in BA (out of ten) were infected with WSMV (Figure 7). 

In contrast to this high infection rate, sRNA HTS failed to detect the presence of WSMV in 2_ECG_US and 3_SVCD_US. In 1_M_US and 6_ECGSV_BA, two and six WSMV-derived contigs were detected, but the number of the normalised redundant reads was below our set threshold (64 and 85 in 1_M_US and 6_ECGSV_BA, respectively) (Table S3). In these two libraries, the coverage of the WSMV genome was just slightly below the set (60%) threshold, being 57.8 and 57.3, respectively (Table S3). The majority of the WSMV-derived reads in 2_M_BA and these two libraries were 21 nt long, suggesting active antiviral silencing (Figure 1). 

**Figure 8:**
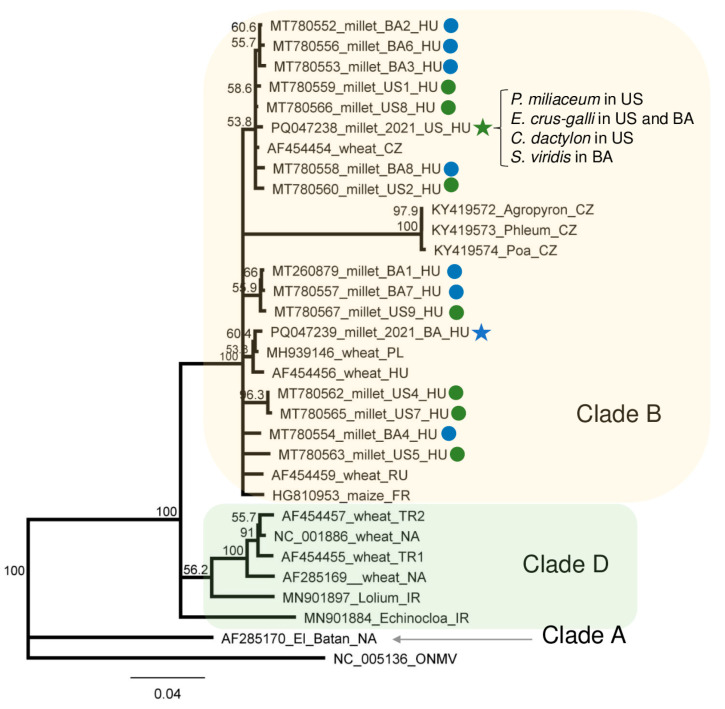
The phylogenetical analysis of the WSMV strains originating from BA and US. The phylogenetic tree was constructed based on the 755 nt long amplified and Sanger-sequenced, polyprotein-encoding (containing CP coding) part of the viral genome using the Neighbour-Joining analysis and the Jukes–Cantor model, with 1000x bootstrap replications. Bars represent 4% nucleotide diversity. Sequences originating from our previous study are marked with circles, while sequences from this study are marked with stars. Green represents US, while blue represents BA. Sequences of the different strains are marked with their GenBank accession numbers, host species, and countries of origin. HU—Hungary, CZ—Czechia; PL—Poland; RU—Russia; FR—France; TR—Turkey; NA—North America; IR—Iran. Green—US, blue—BA. ONMV—Oat necrotic mottle virus, used as an outgroup to root the tree.

Sequencing PCR products originating from the species pool showed the absence of the SNPs, showing no sequence variation within the amplified virus part. That is why only one PCR product amplified from each species pool was cloned and sequenced. Although we sequenced WSMV strains in six cases, we only found two variants (PQ047238_2021_MUS and PQ047239_2021_MBA) (Table S5). The sequence of the MUS strain, present in millet in US, which was found in *E. crus-galli* (both in US and BA), *C. dactylon* (in US), and *S. viridis* (in BA), and the MBA strain present in millet at BA was 98.8% identical (Table S6). They were also very similar (higher than 97% similarity) with the variants sequenced in millet in 2019). Comparing the WSMV strains sequenced in Hungary to other strains, this identity decreased to 81%. However, there are several different WSMV sequences available in the GenBank, some of them originate from another parts of the virus, which is why we could not take them into account during the phylogenetical analysis. The variants sequenced during this study cluster into Clade B with the previously sequenced European variants (Figure 8).

#### 2.2.2. Besides Millet in BA, BYSMV Is Present in *C. dactylon* in US

**Figure 9:**
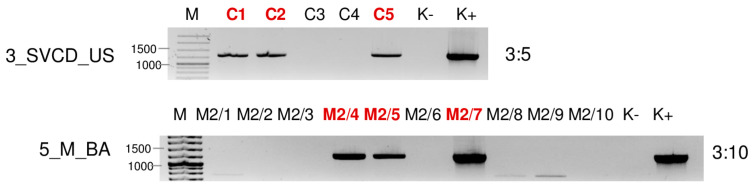
Virus diagnostics using RT-PCR to test the presence of BYSMV in the sampled individuals, amplifying a 1287 bp long part of the BYSMV genome using BYSMV_158F and BYSMV_1444R primers (sequences are available in Table S4). M stands for a GenRuler 100 bpPlus, used as a molecular marker. K− is the negative control, while K+ is the positive control. Red indicates positive individuals The codes of the sRNA HTS libraries and the numbered individuals are marked based on Table A1. The infection rate of the plant species is also indicated.

Testing the presence of BYSMV in the species pools revealed infection of *C. dactylon* in US and millet in BA. While in BA, three millet samples out of ten were infected, testing individuals using RT-PCR showed that out of the tested five *C. dactylon* samples from US, three were infected (Figure 9).

**Figure 10:**
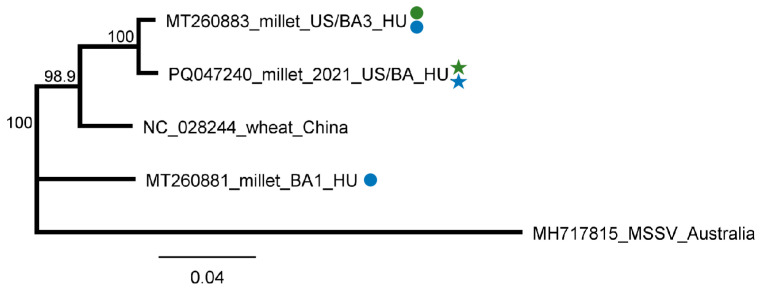
Phylogenetic analysis of the BYSMV strains originating from BA and US. The phylogenetic tree was constructed based on the 1287 nt long amplified and Sanger-sequenced, CP-coding part of the viral genome using Neighbour-Joining analysis and the Jukes–Cantor model, with 1000x bootstrap replications. Bars represent 4% nucleotide diversity. Sequences originating from our previous study are marked with circles, while sequences from this study are marked with stars. Green represents US, while blue represents BA. Sequences of the different strains are marked with their GenBank accession numbers, host species, and countries of origin. HU—Hungary. MSSV—maize sterile stunt virus (MSSV), used as an outgroup to root the tree.

BYSMV infection in millet was reliably diagnosed: 236 virus-derived contigs were present, resulting in 4446 RPM. Moreover, 97.8% of the viral genomes were covered by small RNA reads. In contrast, in 3_SVCD_US, there was no BYSMV-derived contig and the RPM was 61. The only indication that BYSMV could be present in these plants was that BYSMV-originated small RNAs covered 66.3% of the viral genome (Table S3). The size distribution of the sRNA reads showed a peak at 21 nt in the case of 5_M_BA, while in the case of 3_SVCD_US, the majority of the BYSMV-derived reads were 24 nt long, indicating that in this latter case, the antiviral silencing is not very active (Figure 2). The sequences of the PCR-amplified part prepared from the species pools show no differences, and when sequenced as a clone, turned out to be the same (PQ047240_2021_CDUS). It was very similar to (99.78% similarity) and was clustered together with one of the variants found in 2019 at the same place (Table S7) (Figure 10). 

#### 2.2.3. BVG Can Infect *E. crus-galli* and Did Not Induce Strong Antiviral RNAi in Millet

**Figure 11:**
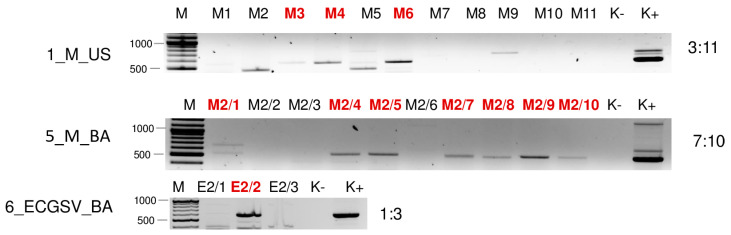
Virus diagnostics using RT-PCR to test the presence of BVG in the sampled individuals. amplifying a 523 bp long part of the BVG genome using BVG_700F and BVG_1223R in cases of 1_M_US and 5_M_BA and a 607 bp long part of the BVG genome using BVG_3508F and BVG_4115R primers in case of 6_ECGSV_BA (sequences are available in Table S4). M stands for a GenRuler 100 bpPlus, used as a molecular marker. K− is the negative control, while K+ is the positive control. Red indicates positive individuals. The codes of the sRNA HTS libraries and the numbered individuals are marked based on Table A1. The infection rate of the plant species is also indicated.

sRNA HTS diagnosed BVG infection in *E. crus-galli* in BA, suggesting this plant is a new host of BVG. This infection was validated using RT-PCR (Figure 6), but a PCR product of the expected size was also amplified from *E. crus-galli* in US and in millet at both locations. The Sanger sequencing of the PCR products revealed that they originated from BVG, with one exception. The appr 600 nt product in the 2_ECG_US sample turned out to be a false positive, as it was revealed to have originated from the plant genome (*P. virgatum* 76%, AC243233), having similarities only in the primer sequence. Searching for this sequence in the *E. crus-galli* sequences available in the GenBank did not result in any hits, but it shows a 98% similarity with sequences from other monocotyledonous species, supporting its background origin. We designed new primers to avoid false positive results during the testing of the individual plants belonging to the positive pools (BVG_707F and BVG_1223R) (Table S4). The RT-PCR of the individuals showed that three and seven millet plants (out of eleven and ten, respectively) and only one *E. crus-galli* plant (out of three) were infected (Figure 11). 

**Figure 12:**
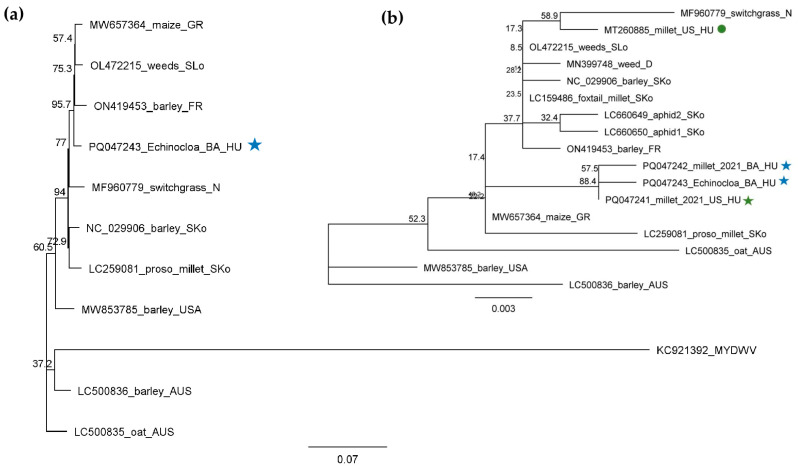
Phylogenetic analysis of the BVG strains originating from BA and US. The phylogenetic tree was constructed based on (**a**) nearly full (5362 nt long) BVG genomes and (**b**) on the 608 bp long amplified and Sanger-sequenced, CP-coding part of the viral genome using the Neighbour-Joining analysis and the Jukes–Cantor model, with 1000x bootstrap replications. Bars represent (**a**) 7% and (**b**) 0.3% nucleotide diversity. Sequences originating from our previous study are marked with circles, while sequences from this study are marked with stars. Green represents US, while blue represents BA. Sequences of the different strains are marked with their GenBank accession numbers, host species, and countries of origin. GR—Great Britain; SLo—Slovenia; FR—France; HU—Hungary; N—the Netherlands; SKo—South Korea; AUS—Australia; D—Germany. MYDWV—Maize yellow dwarf virus, used as an outgroup to root the tree.

In this latter case (6_ECGSV_BA), we found 18 BVG-mapped contigs, the RPM was 212 and the small RNAs covered 90% of the viral genome. In the case of the millet plants, there was no BVG-derived contig; the RPM was very low, namely 14 and 10; and the coverage of the viral genome by small RNA reads was only 33% and 40%, respectively (Table S3). The size distribution of the BVG-derived small RNAs showed that in the case of 6_ECGSV_BA, the majority of the small RNAs were 21 nt long, while in the case of 1_M_US and 5_M_BA, the majority of the small RNAs were 24 nt and 21 nt long (Figure 3). A nearly complete genome of the BVG strain in the *E. crus-galli* was amplified and sequenced (PQ047243). A BVG variant sequenced on *E. crus-galli* is highly similar to the strains available in the GenBank, sequenced in different hosts at different locations. It shows the highest identity to a BVG variant sequenced in Great Britain from maize (98.7%) and in France from barley (98.3%) (Table S8/A). The sequence analysis of the smaller, 607 nt long part of the genome showed a similar, slightly different trend (Table S8/B). The variants sequenced from millet in US and BA and from *E. crus-galli* in BA were highly identical (higher than 99% identity) and were very similar to the variant sequenced in 2019 (higher than 98.6% identity). BVG variants were very similar, and the variants sequenced in Europe showed a higher than 97% identity, suggesting a very conservative genome of the virus. The BVG variant from *E. crus-galli* clustered with the variants present in Great Britain, Slovenia, and France. Although they were similar, the variants sequenced in 2019 and in this study clustered distantly (Figure 12).

#### 2.2.4. ApGlV1 Is Present in Hungary and Was Detected in New Hosts: *S. halepense*, *E. crus-galli*, and *Z. mays*

**Figure 13:**
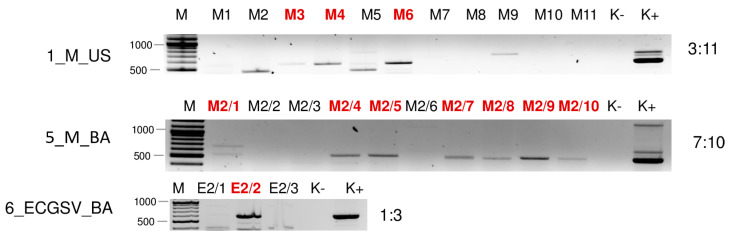
Virus diagnostics using RT-PCR to test the presence of ApGlV1 in the sampled individuals amplifying a 906 bp long part of the ApGlV1 genome using ApGlV1_439F and ApGlV1_1323R primers (sequences are available in Table S4). M stands for a GenRuler 100 bpPlus, used as a molecular marker. K− is the negative control, while K+ is the positive control. Red indicates positive individuals. The codes of the sRNA HTS libraries and the numbered individuals are marked based on Table A1. The infection rate of the plant species is also indicated.

SRNA HTS yielded a hit to ApGlV1 in 7_Ma_BA, revealing the presence of 91 ApGlV1-derived contigs, 2359 RPM, and the 90% coverage of the viral genome. Its presence was validated using primers designed according to the small RNA reads, covering the partial capsid protein-encoding part of the viral genome. Besides *Z. mays*, the ApGlV1-specific PCR product was amplified in 4_SH_U and 6_ECGSV_BA (Figure 6). Infection of the individual plants in each species pool was determined using RT-PCR and showed that three *S. halepense* at Újmajor, two *E. crus-galli*, and two *Z. mays* in BA were infected with the virus (Figure 13). 

SRNA HTS diagnosed infection with ApGlV1 only in maize, where the size distribution of the ApGlV1-derived small RNA reads showed that they were mostly 20 nt long. In 4_SH_U, the reads showed peaks at 21 and 24 nt, while in 6_ECGSV_BA, they were 24 nt long (Figure 4).

**Figure 14:**
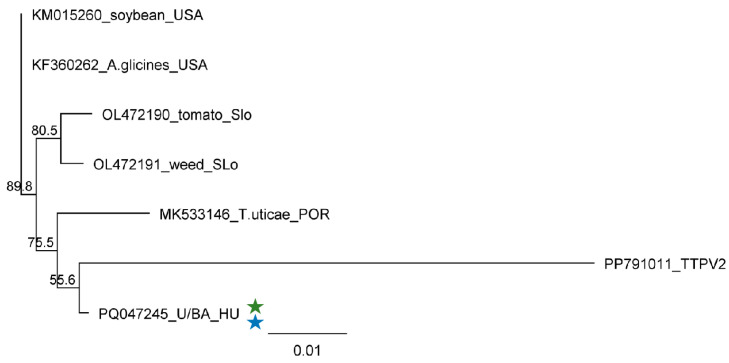
Phylogenetic analysis of the ApGlV1 strains originating from U and BA. The phylogenetic tree was constructed based on the 906 nt long amplified and Sanger-sequenced, VP4/VP3-coding part of the viral genome using the Neighbour-Joining analysis and the Jukes–Cantor model, with 1000x bootstrap replications. Bars represent 1% nucleotide diversity. Sequences originating from this study are indicated with stars. Green represents US, while blue represents BA. Sequences of the different strains are marked with GenBank accession numbers, host species, and countries of origin. SLo—Slovenia; POR—Portugal; HU—Hungary. PP791011—Tetranychus truncatus picorna-like virus 2 (TTPV2), used as an outgroup to root the tree.

The sequencing of the PCR products amplified from the species pools shows no variation. The sequences of the cloned products showed no variation between the strains present in the three different species. The Hungarian variant is very similar to the other sequences, and the sequence identity of all available strains is higher than 98.2% (Table S8). In addition to the high similarity, the variants clustered together according to their geographical origin, irrespective of their plants or insect hosts, indicating their on-site origins (Figure 14). The Hungarian variant clustered distantly to the ApGlV1 variants, together with the sequence of ApGlV1’s closest homologue, namely the Tetranychus truncatus picorna-like virus 2 (TTPV2), which we used as an outgroup. It is interesting to note that considering the amplified part this virus is higher than 92% identical to the ApGlV1 variant, it can be only a divergent strain of the same virus. Although the Hungarian variant was also only 92% identical to TTPlV2, phylogenetically it is more related to this strain than to the other variants.

#### 2.2.5. LDV1 Was Detected for the First Time in Hungary in Several Different Hosts

**Figure 15:**
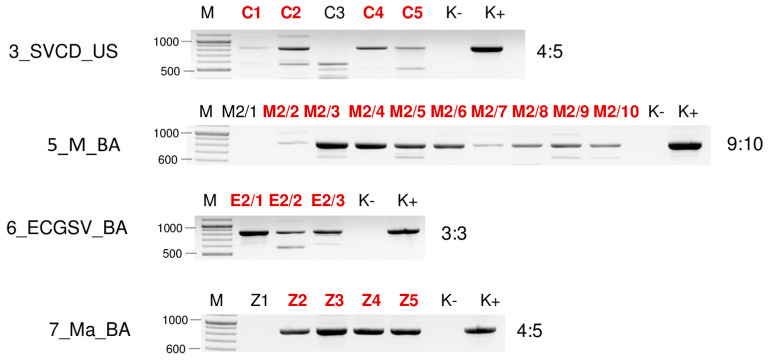
Virus diagnostics using RT-PCR to test the presence of LDV1 in the sampled individuals amplifying an 838 bp long part of the LDV1 genome using LDV1_5664F and LDV1_6480R primers (sequences are available in Table S4). M stands for a GenRuler 100 bpPlus, used as a molecular marker. K− is the negative control, while K+ is the positive control. Red indicates positive individuals. The codes of the sRNA HTS libraries and the numbered individuals are marked based on Table A1. The infection rate of the plant species is also indicated.

SRNA HTS gave results for the presence of LDV1 in 7_Ma_BA. In this library, 1 LDV1-specific contigs and 76% coverage of the viral genome were found, while the RPM was only 87, suggesting, if it even existed, a weak silencing response for the presence of this virus (Table S3). Using our in-house-designed primers, LDV1-specific 816 bp products were amplified in three additional libraries, namely 3_SVCD_US, 5_M_BA, and 6_ECGSV_BA (Figure 6). LDV1 has not been described in any of these plant species, why to further validate the presence of this recently described virus individuals of the species pools were tested for its presence. In the tested species, we found a relatively high infection rate. Four *S. viridis* samples (out of five) in US, nine millet samples (out of ten) in BA, three *E. crus-galli* samples (out of three), and four maize samples (out of five) turned out to be infected (Figure 15). 

**Figure 16:**
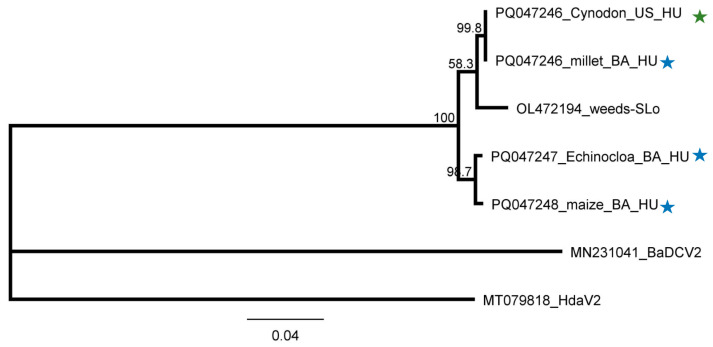
Phylogenetic analysis of the LDV1 strains originating from US and BA. The phylogenetic tree was constructed based on the 838 nt long amplified and the Sanger-sequenced part of the viral genome, encoding the end of the viral RdRP and the beginning of the CP, using the Neighbour-Joining analysis and the Jukes–Cantor model, with 1000x bootstrap replications. Bars represent 4% nucleotide diversity. Sequences originating from this study are indicated with stars. Green represents US, while blue represents BA. Sequences of the different strains are marked with their GenBank accession numbers, host species, and countries of origin. SLo—Slovenia; HU—Hungary. MN231041—Bemisia-associated dicistrovirus 2 (BaDCV2), used as an outgroup to root the tree.

The investigation into the size-distribution of the relatively low amount of LDV1-derived small RNA reads showed that, in 5_M_BA, the majority were 21 nt long; in 3_SVCD_US and 6_ECGSV_BA, the majority were 24 nt long; while in 7_Ma_BA, most of the reads were 20 nt long (Figure 5). Th sequencing of the PCR products revealed no SNPs within the amplified region. We cloned and sequenced these LDV1-derived PCR amplified products and found that they were identical in the cases of *S. viridis* and *P. miliaceum* but slightly different in the other two species. The Hungarian variants were 97.9–99.4% and 95.9–97.8% identical to each other or to the Slovenian variant, respectively. The variants found in BA in *E. crus-galli* and maize clustered together, indicating their common origin (Figure 16).

### 2.3. Revisiting Data from Our 2019 Pilot Survey Looking for the Presence of ApGlV1 and LDV1

In this study, we identified the presence of ApGlV1 and LDV1 in US and BA; moreover, we detected LDV1 in millet. During the analysis of the samples collected in 2019, we did not consider the presence of either ApGlV1 or LDV1, so they could have been overlooked. To investigate the presence of these viruses in the 2019 collected samples, we reanalysed the 2019 pilot study FASTQ files for their presence, resulting in no hits (no hits through the BLASTN of the contigs or by mapping the redundant reads to the viral genome) (Table S11). As in the current study, because sRNA HTS failed to detect their presence in some cases, an RT-PCR test of the pools representing the sequencing libraries was also carried out; this resulted in no amplified products at the expected size, suggesting that they were not present in the millet sampled in 2019.

### 2.4. Symptoms of the Sampled Plants Cannot Be Explained by the Presence of the Viruses

During our survey, we randomly sampled 56 plants showing virus-like symptoms (Figure S2a–i). SRNA HTS combined with RT-PCR revealed the high infection rate in the plants, revealing virus infections in 37 of them. While 18 plants were infected with a single virus, 19 were infected with several viruses (8, 9, and 2 plants were infected with 2, 3, and 4 viruses, respectively). The infection rate in plants from US was 46% (14 plants out of 30), 60% in U (3 plants out of 5), and 95% in BA, where only 1 plant out of 21 was not infected with any of the viruses and 13 were coinfected. In US, only WSMV, BVG, and ApGlV1 were present; in U the sampled *S. halepense* plants were infected only with ApGlV1; while in BA all five viruses were detected.

Investigating the virus-specific symptoms on the plants did not coincide with their infection status. We sampled symptomatic plants, but 19 of them turned out to be not infected. Plants infected with several viruses did not show stronger symptoms than the non-infected ones (Figure S2a–i).

## 3. Discussion

The small RNA HTS of the rural samples revealed the presence of five viruses in the investigated plants. Three of the hits, namely WSMV, BYSMV and BVG, were viruses found two years earlier at the same locations. In the sRNA HTS-positive libraries, the presence of these viruses was clear, and the high number of the viral reads were evenly distributed on the viral genome. Their size was predominantly 21 nt, reflecting active antiviral DICER (DCL2 and DCL4) activity [40]. The presence of ApGlV1 and LDV1 was found only because we used comparisons, including recently described viruses from Slovenian rural samples [9]. In that study, the sequences of 165 viral strains, representing different viruses originating from Slovenia, were determined and deposited into the GenBank, allowing us to search for their presence with a simple workflow method.

sRNA HTS result did not coincide with the RT-PCR validation and revealed infections in more cases than the sRNA HTS itself (Table 1).

During our sRNA HTS diagnostics, the sRNA sequencing libraries were prepared from pools containing a mixture of individual plant extracts. In this case, the RNA of the non-infected plant could dilute the vsiRNA concentration to below the sensitivity level, leading to a false negative result. However, the infection rate in the investigated cases was not low, which could not explain the false results. 

We found the same situation in the cases of the rupestris stem pitting-associated virus and the grapevine virus T when we investigated virus infections in grapevines [41,42]. In these cases, we hypothesised that the antiviral RNA response has been weakened during long plant–virus coexistence, leading to their latency and the absence of a strong host response, making sRNA HTS unable to detect their infection. This could have happened in some of the current cases. In addition, the type of the virus infection can change over time and is influenced by ecological factors, like changing climate and the behaviour of the vectors [1]. Moreover, virus infection occurs through different phases. At the beginning, the virus infection interferes with the host metabolism, inducing strong symptoms, but can later achieve a persistent state and become latent for a long time [3]. SRNAs are produced in large numbers by the host’s antiviral RNAi during the acute phase of the infection. When the infection becomes persistent, the activity of this defence mechanism and, consequently, the number of the vsiRNAs decrease. This decreased amount of sRNAs is below the sensitivity level of sRNA HTS, which leads to the failed detection of persistent viruses. We think that the false detection of the viruses could be explained by this hypothesis.

In five of the seven sequenced libraries, WSMV was found in four plant species at two locations. Besides the well-described cereal hosts, using serological methods, WSMV was detected in several monocotyledonous species [14], including *C. dactylon* [43], *E. crus-galli*, and *S. viridis* [44]. Furthermore, it was shown that, in Czechia, forced infection in the field was successful in the case of *E. crus-galli* [20]. In addition to these works, WSMV was identified in other hosts using nucleic acid-based detection methods (Table S1), including *E. crus-galli* in Iran (Matsumi 2022, only GenBank records, but this report is the first to provide sequence data from WSMV hosted by *C. dactylon* and *S. viridis*).

During our pilot survey, we found that the WSMV infection rate in US was higher than in BA. This time, the infection rates of the millet plants at the two locations were about the same (3:11 in US and 4:10 in BA). In contrast, if present, the infection rate with WSMV was very high in the grass species. All individuals of *C. dactylon* in US and all three sampled *S. viridis* plants in BA were WSMV-infected, while five (out of eight) and all three *E. crus-galli* were infected in US and BA, respectively. In our previous study, we sequenced several different strains, while only two variants were found this time. They clustered in Clade B with strains sequenced from wheat hosts from Hungary, Czechia, Russia [15], and France [45], and WSMV was sequenced from different weed species in Czechia [46]. The sequences of the variants found in millet in US, *E. crus-galli*, *C. dactylon*, and *S. viridis* were identical and clustered with several WSMV variants from our previous study. The sequence of the WSMV variant sequenced from *E. crus-galli* in Iran is also available and clustered in Clade D, which is characteristic of the Asian (Turkey) variants, with another WSMV variant identified from the same country but from a *Lolium* host, suggesting that the variants do not show host specificity but similarities reflects their geographical origins. The variant present in millet from BA clustered distantly from the variants sequenced in 2019; its closest neighbours were strains sequenced in Poland and Hungary more than two decades ago [15]. At 5_M_BA, sRNA HTS could successfully detect the infection of WSMV, suggesting a new, active infection. In contrast to the other cases, the amount of WSMV-derived siRNA was low, suggesting a balanced virus titer in the plant without active antiviral silencing. In 2_ECG_US and 3_SVCD_US, besides the lack of WSMV-specific contigs, the presence of few virus-derived sRNAs and the low coverage of the viral genome coincided with a range in the sizes of small RNAs, suggesting the low activity of the RNAi in these cases (Table S3 and Figure 1). We think that in the millet plants from BA, a new infection ocurred, while in the other plants, a lower-level background infection was maintained in the overwintering plants. However, the fact that WSMV has been found in different grasses supports our original hypothesis that they can help the virus persist and have a virus reservoir role.

BYSMV was identified in two hosts, namely millet and *C. dactylon*, at the two sampled locations. To our knowledge, this is the first time *C. dactylon* has been identified as a host of this virus. SRNA detected BYSMV only in the 5_M_BA, where the number of BYSMV-derived sRNAs was high, covering the entire genome. Their predominantly 21 nt long size suggests that they are of DCL-2 and DCL-4 origin (Figure 2). In contrast, BYSMV infection in 3_SVCD_US was missed using sRNA HTS. Although they covered the BYSMV genome, the low number of the sRNA reads coincided with their spread-out size distribution, showing the persistent state of the infection in this case. The sequences of the variants found at both locations were the same and were very similar to one of the variants sequenced during our previous study. At that time, another variant, present in BA in two millet plants, was also identified. It is possible that the infections of these strains from 2019 were persistently retained in US in the overwintering roots of *C. dactylon*, not inducing a strong silencing signal in 2021, while the millet in BA was newly infected, resulting in an acute infection. However, this theory should be investigated in the future. *C. dactylon* is an invasive weed, and the presence of a persistent form of BYSMV in it is alarming, as this could serve as a constant source of new virus inoculum.

Besides millet, BVG has been identified as being able to infect *E. crus-galli*, which to the best of our knowledge is a previously undescribed host of the virus. While sRNA HTS could detect the infection only in this latter case, a low infection rate was detected in millet at both locations. Although in 6_ECGSV_BA only one plant out of three was infected, the BVG genome was covered with virus-derived small RNAs, which were predominantly 21 nt long, suggesting a strong antiviral RNAi in this case (Figure 3). In contrast, although several plants (three out of ten in US and seven out of eleven at BA) were BVG-infected, sRNA HTS could not detect its presence, suggesting that the initial acute infection became persistent without inducing a strong RNAi. This time, we found three slightly different BVG variants, which clustered together, suggesting their common origin. Their close identity to the variant sequenced in 2019 from the same location suggests the persistent presence of BVG in the millet population; however, its new introduction is better supported because of its distant clustering. Most reports about BVG are short notes without any information about the induced symptoms but suggest its diverse host range and widespread distribution. BVG was identified in a 34-year-old sample in Australia [47] and in weeds in Germany (we do not have information about the particular host) and Slovenia (in a pooled sample containing *Picris echioides*, *Pastinaca sativa*, and *S. halepense* RNA [9], where we believe that the host was the *Sorghum*). BVG could be present in several places for a long time as a latent infection, and perennial and overwintering monocotyledonous weeds can play an important role in its maintenance. Although we cannot conclude anything about the symptoms induced by BVG itself, it could interfere with other presenting viruses, accelerating their deteriorating effect. BVG encodes P0 protein, identified in other *Poleroviruses* as a viral silencing suppressor, destabilizing the antiviral Argonaute proteins [48,49]. It is possible that during coinfection, the decreased effector activity of this destabilized AGO does not allow efficient host defence; a question which would be interesting to address in future research.

ApGlV1 was found to infect three different plant species, namely *S. halepense*, *E. crus-galli* and *Z. mays*, at two locations. We found only one variant of the virus, similar to other ApGlV1 sequences from insects or plants (soybean or tomato), suggesting a very high conservation of its genome. Infection with ApGlV1 seems to be persistent in *S. halepense* and *E. crus-galli*, but acute in the case of maize, reflected by the presence of a high number of vsiRNAs covering the entire genome. The size distribution of the viral reads shows a maximum of 20 nt, which we cannot rationalise as we do not know any plant DICER with this type of activity (Figure 4). 

LDV1, a recently identified dicistrovirus, was found in four plant species at both locations, revealing *C. dactylon*, *P. miliaceum*, *E. crus-galli*, and *Z. mays* as new hosts of the virus. We found three slightly different variants, suggesting onsite evolution. The majority of the vsiRNAs were 21 nt long in the case of the millet samples, which is characteristic for the acute phase of an infection with an active RNAi, while in the other species, the infection seemed to reach a persistent state. It is interesting to note that the size distribution of the vsiRNAs showed a maximum length at 24 nt in *C. dactylon* and *E. crus-galli*, while the majority were 20 nt long in maize (Figure 5). The same characteristics found in the case of ApGlV1 in the same plant raise the possibility that if they are not technical byproducts, these dicistroviruses are processed in maize in a slightly altered way to in canonical plant viruses; however, to answer this question, further experiments would be needed. LDV1 was found to infect plants, but being a dicistrovirus, which is usually present in insect vector species, we think it is present and distributed by insect vector species. ApGlV1 and LDV1 were identified recently, and there is no information about their possible impact on host fitness; a question that is also open for further research.

The reinvestigation of our 2019 pilot study fatsq files for the presence of ApGlV1 and LDV1 resulted in no hits. Moreover, we could not detect these two viruses through RT-PCR in the samples collected in 2019, showing that ApGlV1 and LDV1 were not present in the millet sampled in 2019, suggesting that the virus was not present in US or BA in 2019. However, as we sampled only twenty plants, we cannot exclude the possibility that they were present there, just not in the investigated plants.

We found no correlation between the presence of the viruses and the observed symptoms. Most of the presenting viruses could have reached the persistent state of their infection and become latent, subsequently becoming influenced by several different abiotic factors, which could result in virus-like symptoms.

We found a striking difference between the infection rates of the sampled locations. US was infected only with three viruses: WSMV, BVG, and ApGlV1, with the latter virus only found in *C. dactylon*. At this location, wheat and maize were cultivated alternatively, and in the year of sampling, wheat was grown and was harvested just before the sample collection. In U, where no intensive cultivation was carried out, we only sampled an established population of *S. halepense*, which was infected by ApGlV1 only. BA was highly infected with viruses, and we found the plants to be coinfected in several cases. Here, maize was cultivated, and we also found viruses in the crops. This location was at the edge of a small creek where many plants grew extensively on its banks, offering a more humid environment and alternative shelter for the vectors. Beside acute infections (WSMV in 1_M_US, 5_M_BA, and 6_ECGSV_BA; BYSMV in 5_M_BA; BVG in 6_ECGSV_BA; ApGlV1; and LDV1 in 7_Ma_BA), we found several cases of persistent infections, suggesting possibilities for the virus reservoir roles of these plants. 

## 4. Materials and Methods

### 4.1. Plant Material and Total Nucleic Acid Extraction

Leaf samples of *Panicum miliaceum*, *Zea mays*, and monocotyledonous weeds, namely *Echniocloa crus-galli*, *Cynodon dactylon*, *Setaria viridis*, and *Sorghum halepense* (56 sampled plants in total), were collected in June 2021 at three different locations: Újmajor susnyás, Újmajor, and Büdös árok (see Table A1 for detailed information and Figures S1 and S2a–i for photos of the sampled individuals). Several individuals of the same species, showing different symptoms, were sampled randomly. Total nucleic acid was extracted from the frozen leaves using a phenol–chloroform method [13]. Briefly, frozen plant material was homogenized in an ice-cold mortar in 650 µL of extraction buffer (100 mM of glycine, pH 9.0, 100 mM of NaCl, 10 mM of EDTA, 2% SDS, and 1% sodium lauroylsarcosine), mixed with an equal volume of phenol, and centrifuged for 5 min. The aqueous phase was added to equal volumes of phenol, chloroform, and isoamyl alcohol (25:24:1) and centrifuged for 5 min. The upper phase was added to an equal volume of chloroform/isoamyl alcohol (24:1) and centrifuged for 5 min. The upper phase was precipitated with ethanol and washed with 70% ethanol. After centrifugation, the resulting pellet was dried and then re-suspended in sterile water. The RNA quality, in terms of integrity, was checked using agarose gel electrophoresis. The obtained total nucleic acid extracts were stored at −70 °C until used.

### 4.2. Preparation of the sRNA Sequencing Libraries and Sequencing

RNA pools were prepared by mixing equal amounts of total nucleic acid from individuals of the same species (in the cases of five libraries) and from two different species (in the case of 3_SVCD_US and 6_ECGSV_BA) originating from the same location. Retaining the individual total nucleic extracts and this pooling strategy allowed us to detect the presence of any virus present in any of the sampled individual plants. The pools were used alone or in combination for the small RNA sequencing library preparation (seven libraries in total), as detailed in Table A1. The SRNA fractions of the pools were separated on a polyacrylamide gel and purified following our in-house updated protocol [50] based on the TruSeq Small RNA Library Preparation Kit (Illumina, San Diego, CA, USA). Seven sRNA libraries were sequenced using a single index on a HiScanSQ by UD-Genomed (Debrecen, Hungary) (50 bp, single-end sequencing). The FASTQ files of the sequenced libraries were deposited in the GEO and can be accessed through series accession number GSE270076.

### 4.3. Bioinformatic Analysis of the HTS Results

The FASTQ files of the HTS were analysed using the CLC Genomic Workbench (Qiagen, Hilden, Germany). After the trimming and quality control of the reads, longer contigs were built from the non-redundant reads using a CLC assembler (de novo assembly using default options, namely word size 20 and bubble size 50, and simple contig sequences and min. 35 nt length) (see Table S2 for the initial statistics of the reads). The contigs were annotated using the BLASTN algorithm with default options (thread 1, word size 11, match 2, mismatch 3, gap cost existence 5, extension 2) using the NCBI Plant-Hosted Viral Reference genomes (downloaded at 5 December 2023) and a collection of 165 virus sequences sequenced in Slovenia from rural samples [9]. In the case of viruses represented by at least one contig, the reads were directly mapped to the reference genome and counted with and without redundancy (using the map to the reference command allowing one mismatch). The number of normalized redundant reads (read/1 million reads—RPM) was calculated from the mapped redundant reads and the number of total sequenced reads. Based on the mapping, a consensus sequence was prepared and used to calculate the coverage (%) of the viral genome (see all of the calculations in Table S3). The coverage of the viral genome by the sequenced sRNAs was prepared in all cases when the virus presence was detected by RT-PCR, irrespective of the result of the initial bioinformatic analysis. The size distribution of the virus-mapped reads was also calculated using the QC report command in the CLC Genomic workbench. If at least two parameters of any of (i) the presence of any virus-specific contigs, (ii) the number of normalized redundant virus-specific reads of >200, or (iii) the coverage of the virus genome of >60% were fulfilled, we further investigated the presence of the virus first by reannotating the viral-specific contigs and then by RT-PCR, an independent virus diagnostic method.

### 4.4. Validation of the HTS by RT-PCR

Pooled RNA extracts representing each species pool (nine pools in total) and total nucleid acid extracts of each plant were used as templates for cDNA synthesis with a RevertAid First Strand cDNA Synthesis Kit (Thermo Fisher Scientific, Waltham, MA, USA) and random primers according to the manufacturer’s instructions. For RT-PCR, we used Q5 Hot Start High-Fidelity DNA Polymerase (New England Biolabs, Ipswich, MA, USA) (the primers used to amplify viral parts together with the used annealing temperatures and cycling parameters are provided in Table S4). cDNA prepared from the positive pools of both our previous survey and this article was used as a positive control; cDNA was supplemented by water in the negative controls. PCR products were purified using the GeneJET Gel Extraction Kit (Thermo Fisher Scientific), cloned into GeneJET (Thermo Fisher Scientific), and Sanger-sequenced (ordered as a service). Sequences were deposited into GenBank (PQ047238–PQ047248).

### 4.5. Phylogenetic Analysis of the Detected Viral Strains

Multiple sequence alignments were conducted using Geneious prime and the MUSCLE algorithm. The evolutionary history was inferred using the Jukes–Cantor model and the Neighbour-Joining method. The trees were constructed using the best fit model for each alignment, with 1000 bootstrap replicates. The trees were drawn to scale, with branch lengths measured in the number of substitutions per site. For the outgroups, we used the closest relative of the virus, outlined in the tree’s legend.

## 5. Conclusions

Using sRNA HTS for the virus diagnostics of the weeds was not the best choice—using it on its own meant that it was easy to overlook persistent infections, because the inactive silencing did not produce enough sRNAs to reach its sensitivity level. However, combining sRNA HTS with RT-PCR turned out to be sensitive enough. This combination was able to differentiate between acute and persistent infection and suggest information about the history of the virus infections and their coexistence in several hosts. We found a high rate of persistent-phase infections in weeds, suggesting that the virus has been present in the plant for a long time. Our study identified the presence of viruses at new locations and hosts of previously known viruses, which had not yet been described, thus widening our knowledge of the global viromes in different ecosystems. Although some of the investigated plants are annual weeds, they can act as reservoirs during vegetation periods; however, the virus reservoir role of the weeds can result in infection in the presence of vectors. Further studies in this direction would not only help us to understand the virus flow in natural ecosystems but also help us to find and plan efficient protection strategies to avoid economically important yield losses in the future.

## Figures and Tables

**Table 1:** Summary of the sRNA HTS and RT-PCR diagnostics. The codes of the sRNA HTS libraries are marked based on Table A1. Positive hits are marked with green. The infection rate of the plant species is also indicated.

Library	Test	WSMV	BYSMV	BVG	ApGlV1	LDV1
**1_M_US**	**sRNA HTS**	–	–	–	–	–
	**RT_PCR**	3:11	–	3:11	–	–
**2_ECG_US**	**sRNA HTS**	–	–	–	–	–
	**RT_PCR**	5:8	–	–	–	–
**3_SVCD_US**	**sRNA HTS**	-	-	-	-	-
	**RT_PCR**	5:11	3:11	-	-	4:11
**4_SH_U**	**sRNA HTS**	-	-	-	-	-
	**RT_PCR**	-	-	-	3:5	-
**5_M_BA**	**sRNA HTS**	+	+	-	-	-
	**RT_PCR**	4:10	3:10	7:10	-	9:10
**6_ECGSV_BA**	**sRNA HTS**	-	-	+	-	-
	**RT_PCR**	6:6	-	1:6	2:6	3:6
**7_Ma_BA**	**sRNA HTS**	-	-	-	+	+
	**RT_PCR**	-	-	-	3:5	4:5

## Data Availability

FASTQ files of the sequenced libraries were deposited in the GEO and can be accessed through series accession number GSE270076. Partial viral sequences of the viral were deposited into GenBank (PQ047238–PQ047248).

## Supplementary Materials

The following supporting information can be downloaded at: https://www.mdpi.com/article/10.3390/plants13182664/s1. Figure S1: Map of the geographical location, together with a photo of the sampling area. Figure S2: Photos of the sampled individuals, indicating their registration number. Table S1: List of different hosts of WSMV, BYSMV, BVG, ApGlV, and LDV1, supported by GenBank records. Table S2: Initial statistics of the sequenced small RNA libraries. Table S3: Summary of the bioinformatics analysis. Table S4: Sequences of the PCR primers used for virus detection, cloning, and Sanger sequencing. Table S5: Summary of the viral sequences deposited into the NCBI GenBank. Table S6: Percent identity matrix of the WSMV sequences which were considered in the phylogenetic analysis. Table S7: Percent identity matrix of the BYSMV sequences which were considered in the phylogenetic analysis. Table S8: Percent identity matrix of the BVG sequences which were considered in the phylogenetic analysis. Table S9: Percent identity matrix of the ApGlV1 sequences which were considered in the phylogenetic analysis. Table S10: Percent identity matrix of the LDV1 sequences which were considered in the phylogenetic analysis. Table S11: Summary of the bioinformatics analysis investigating the presence of ApGlV and LDV1 in the 2019 millet samples.

## Appendix A

**Table A1:** Basic information about the sampled plants including a description of the sRNA library codes, sampled plant species name, pooling strategy, symptoms, and detected viruses. Abbreviations used for viral symptoms are: Ldef—leaf deformation; M—mosaic; MM—mild mosaic; Chl—chlorosis; MC—mild chlorosis; Mo—mottle; P—purple coloration; N—necrosis; TN—tip necrosis; VN—vein necrosis; Stu—stunting; TN—tip necrosis.

sRNA HTS Library Code	Place of Sampling	Sampled Plant Species	Code of the Individual Plant	Symptoms	Detected Viruses
**1_M_US**	Újmajor susnyás/ wheat field in 2022	*Panicum miliaceum*	M1	Ldef, M, Chl	
			M2	M, Ldef	
			M3	M, Ldef, P	BVG
			M4	M	BVG
			M5	M, MC	
			M6	M, Ldef	WSMV, BVG
			M7	M, Chl, N, Stu, Ldef	
			M8	M, Chl	
			M9	M, Chl, Ldef	
			M10	M, Chl, TN	WSMV
			M11	M, TN	
**2_ECG_US**		*Echinocloa crus-galli*	E1		WSMV
			E2	Ldef, Stu	WSMV, BVG
			E3	Ldef, Chl, Stu	WSMV, BVG
			E4	Ldef, MM, Chl, Stu	
			E5	Ldef, Chl, N, Stu, M	WSMV
			E6	Ldef, Stu, Chl, M	
			E7	Chl,M,Ldef	WSMV
			E8	Ldef,Stu,N, Chl,M	
**3_SVCD_US**		*Setaria viridis*	S1	M, Ldef, N	
			S2	VN,Ldef, P	-
			S3	M, Ldef, P, Chl	
			S4	Ldef, Chl, P	
			S5	M, N, TN, Ldef	
			S6	Ldef, P, TN, Chl	
		*Cynodon dactylon*	C1	Ldef, M, Mo	WSMV, BYSMV, LDV1
			C2	Ldef, M, Mo, Chl, TN	WSMV, BYSMV, LDV1
			C3	Ldef, M, Mo, Chl, TN	WSMV
			C4	Ldef, MM, Mo	WSMV, LDV1
			C5	MM, TN	WSMV, BYSMV, LDV1
**4_SH_U**	Újmajor/ no crop in 2022	*Sorgum halepense*	H1	M, Ldef, TN	
			H2	M, P	ApGlV1
			H3	M, Stu, Ldef	
			H4	Ldef, M, Stu, P	ApGlV1
			H5	M, Stu, P	ApGlV1
**5_M_BA**	Büdös árok/ corn field in 2022	*Panicum miliaceum*	M2/1	M, Chl, Ldef	BVG
			M2/2	Stu, Chl, M, Ldef	LDV1
			M2/3	Chl, N, Stu, Ldef	LDV1
			M2/4	Stu, Chl, M	BYSMV, BVG, LDV1
			M2/5	Chl, Ldef, M, N	WSMV, BYSMV, BVG, LDV1
			M2/6	Stu, Chl, M, TN	WSMV, LDV1
			M2/7	Chl, M, Ldef, P, N, Stu	WSMV, BYSMV, BVG, LDV1
			M2/8	M, Ldef	WSMV, BVG, LDV1
			M2/9	Stu, M, N	BVG, LDV1
			M2/10	M, Chl, N, Mo	BVG, LDV1
**6_ECGSV_BA**		*Echinocloa crus-galli*	E2/1	Stu, Ldef, M	WSMV, ApGlV1, LDV1
			E2/2	Ldef, Stu, Chl	WSMV, BVG, LDV1
			E2/3	M, Ldef	WSMV, ApGlV1, LDV1
		*Setaria viridis*	S2/1	M	WSMV
			S2/2	M, Ldef, TN, Chl	WSMV
			S2/3	M	WSMV
**7_Ma_BA**		*Zea mays*	Z1	Chl, M, Ldef	
			Z2	M, Stu, Chl	ApGlV1, LDV1
			Z3	Chl, M, Stu	ApGlV1, LDV1
			Z4	Stu	LDV1
			Z5	Chl, Stu	LDV1
