# Supplementary material for: Viromes of Monocotyledonous Weeds Growing in Crop Fields Reveal Infection by Several Viruses Suggesting Their Virus Reservoir Role
Source: Plants (Basel). 2024 Sep 23;13(18):2664. doi: 10.3390/plants13182664 (PMC11435186; doi:10.3390/plants13182664)
Supplement: Supplementary file 1 [file plants-13-02664-s001.zip › Galbacs _etal_SFigures.pdf]

plants

MDPI

Supplementary Figures to

Virome of monocotyledonous weeds growing at crop fields revealed infection with several viruses and suggests their virus reservoir role

Zsuzsanna, N. Galbács[1#], Evans Duah Agyemang[2#], György Pásztor[2], András Péter Takács[2], Éva Várallyay[1*]

[#] These authors equally contributed to the work
*Correspondence: Varallyay.Eva@uni-mate.hu

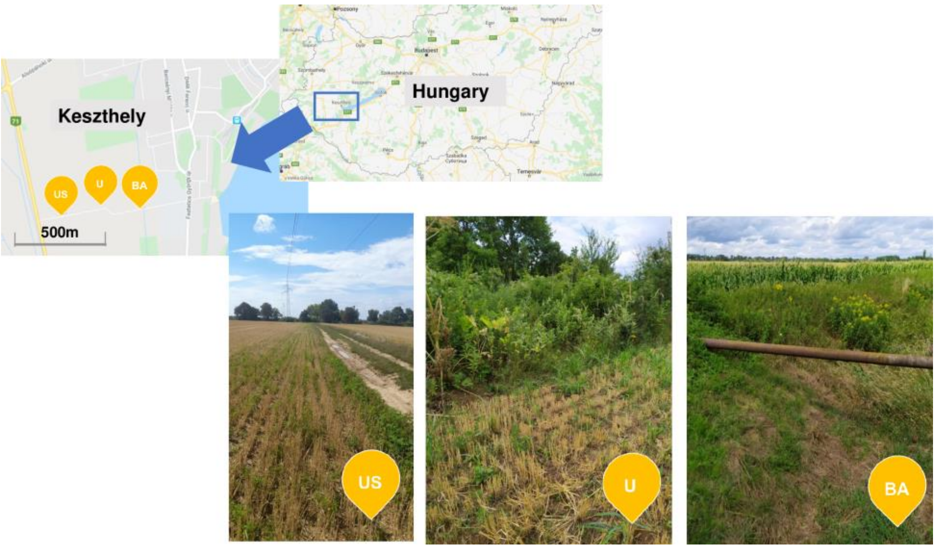

Figure S1. Map of the geographical location, together with a photo of the sampling area

(a)

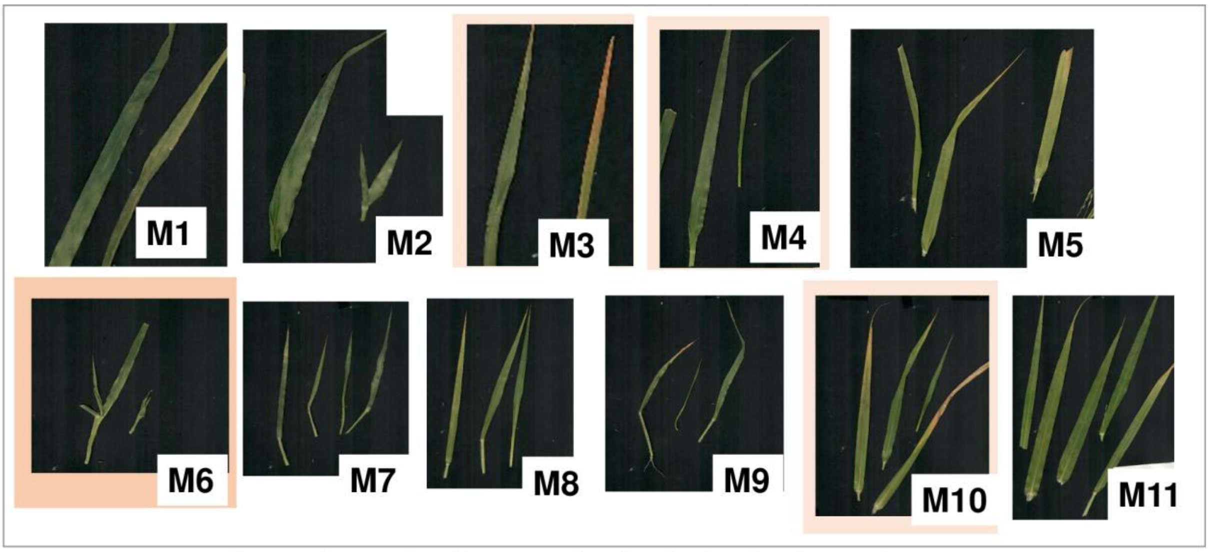

Name of the sampled plant	sRNA HTS library		Symptoms	WSMV	BYSMV	BVG	ApGIV	LDV1	n of infecting viruses
Panicum miliaceum	1_M_US	M1	Ldef, M, Chl						0
		M2	M, Ldef						0
		M3	M, Ldef, P: Purple			1			1
		M4	Mosaic symptoms			1			1
		M5	Mosaic, Mild chlorosis						0
		M6	Mosaic, deformation	1		1			2
		M7	M, Chl, N, Stu, Ldef						0
		M8	M, Chl						0
		M9	M, Chl, Ldef						0
		M10	M, Chl, TN	1					1
		M11	M, TN						0

(b)

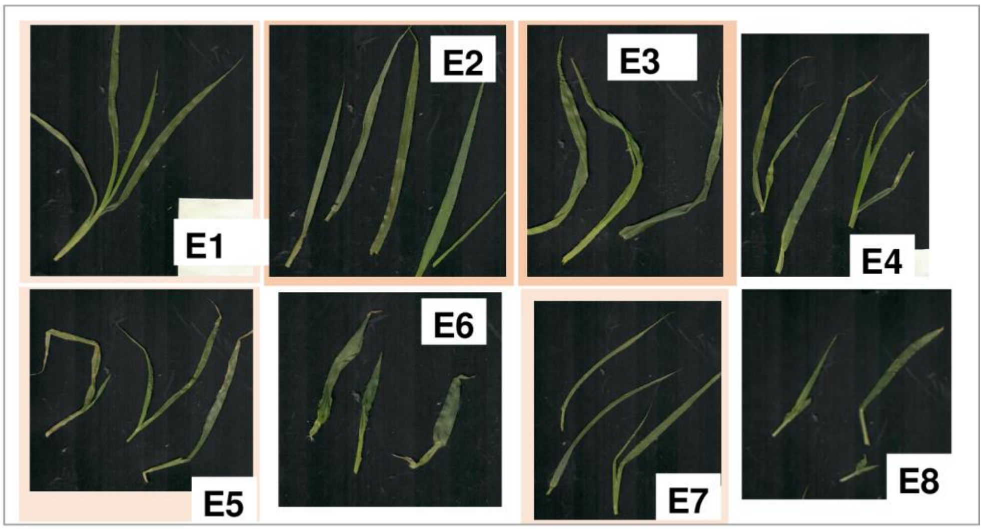

Name of the sampled plant	sRNA HTS library		Symptoms	WSMV	BYSMV	BVG	ApGIV	LDV1	n of infecting viruses
Echinocloa crus-galli	2_ECG_US	E1		1					1
		E2	Ldef, stunting	1		1			2
		E3	Ldef, Chl, stunting	1		1			2
		E4	Ldef, MM, Chl, Stu						0
		E5	Ldef, Chl, Necrosis, stu,	1					1
		E6	Ldef, Stu, Chl, M						0
		E7	Chl,M,Ldef	1					1
		E8	Ldef,Stu,N, Chl,M						0

(c)

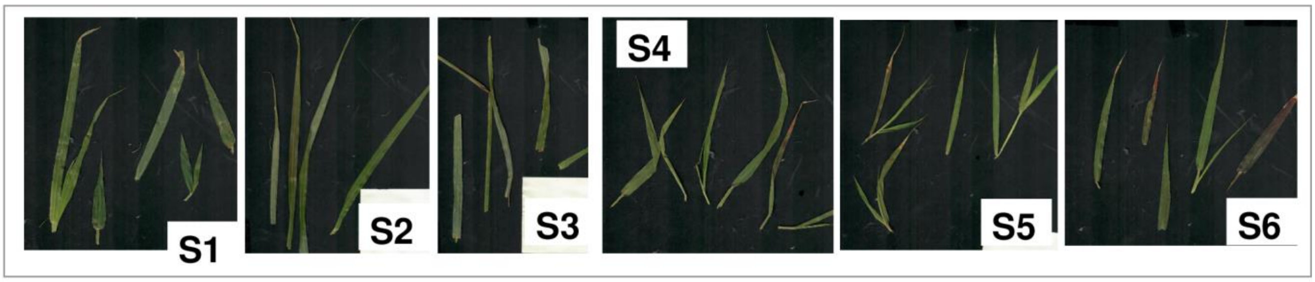

Name of the sampled plant	sRNA HTS library		Symptoms	WSMV	BYSMV	BVG	ApGIV	LDV1	n of infecting viruses
Setaria viridis	3_SVCD_US	S1	M, Ldef, N						0
		S2	vein necrosis,Ldef, Purple coloration						0
		S3	M, Ldef, P, Chl						0
		S4	Ldef, Chl, P						0
		S5	M, N, TN, Ldef						0
		S6	Ldef, P, TN, Chl						0

(d)

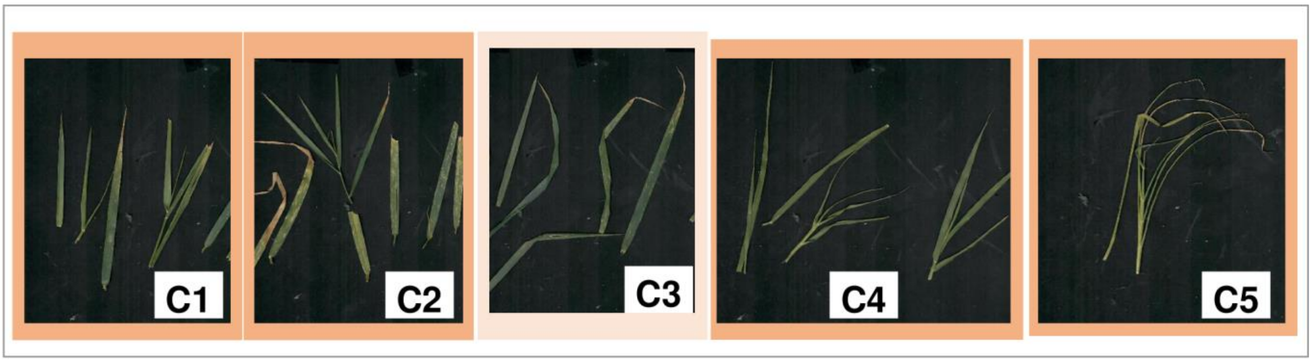

Name of the sampled plant	sRNA HTS library		Symptoms	WSMV	BYSMV	BVG	ApGIV	LDV1	n of infecting viruses
Cynodon dactylon	3_SVCD_US	C1	Ldef, Mosaic, Mottle	1	1			1	3
		C2	Ldef, M, Mo, Chl, TN	1	1			1	3
		C3	Ldef, M, Mo, Chl, TN	1					1
		C4	Ldef, MM, Mo	1	1			1	3
		C5	Mild Mosaic, TN	1	1			1	3

(e)

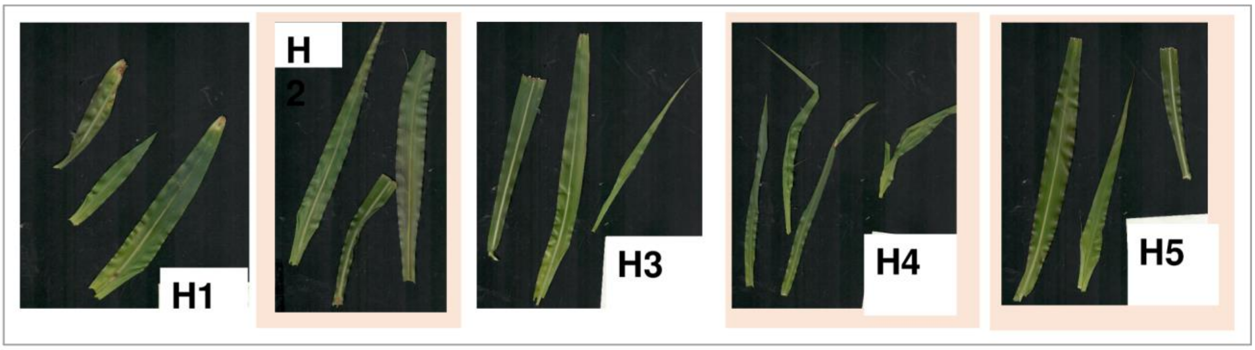

Name of the sampled plant	sRNA HTS library		Symptoms	WSMV	BYSMV	BVG	ApGIV	LDV1	n of infecting viruses
Sorgum halepense	4_SH_U	H1	M, Ldef, TN						0
		H2	M, P				1		1
		H3	M, Stu, Ldef						0
		H4	Ldef, M, Stu, P				1		1
		H5	M,Stu, P				1		1

(f)

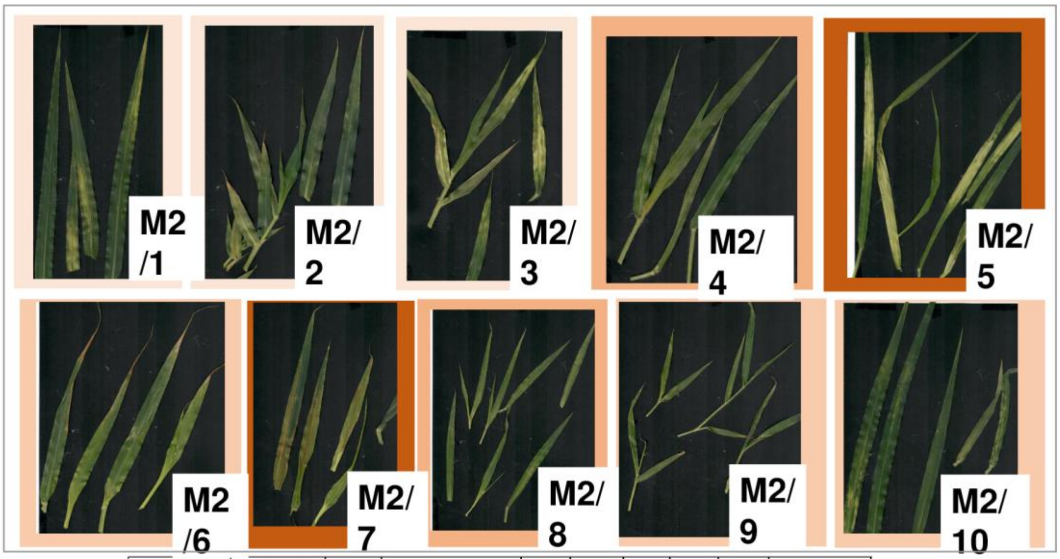

Name of the sampled plant	sRNA HTS library		Symptoms	WSMV	BYSMV	BVG	ApGIV	LDV1	n of infecting viruses
Panicum miliaceum	5_M_BA	M2/1	M, Chl, Ldef			1			1
		M2/2	Stu, Chl, M, Ldef					1	1
		M2/3	Chl, N, Stu, Ldef					1	1
		M2/4	Stu, Chl, M		1	1		1	3
		M2/5	Chl, Ldef, M, N	1	1	1		1	4
		M2/6	Stu, Chl, M, TN	1				1	2
		M2/7	Chl, M, Ldef, P, N, Stu	1	1	1		1	4
		M2/8	M, Ldef	1		1		1	3
		M2/9	Stu, M, N			1		1	2
		M2/10	M, Chl, N, Mo			1		1	2

(g)

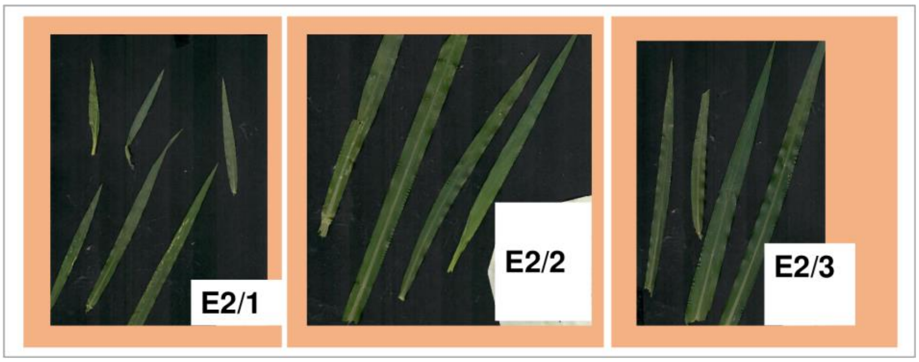

Name of the sampled plant	sRNA HTS library		Symptoms	WSMV	BYSMV	BVG	ApGIV	LDV1	n of infecting viruses
Echinocloa crus-galli	6_ECGSV_BA	E2/1	Stu, Ldef, M	1			1	1	3
		E2/2	Ldef, Stu, Chl	1		1		1	3
		E2/3	M, Ldef	1			1	1	3

(h)

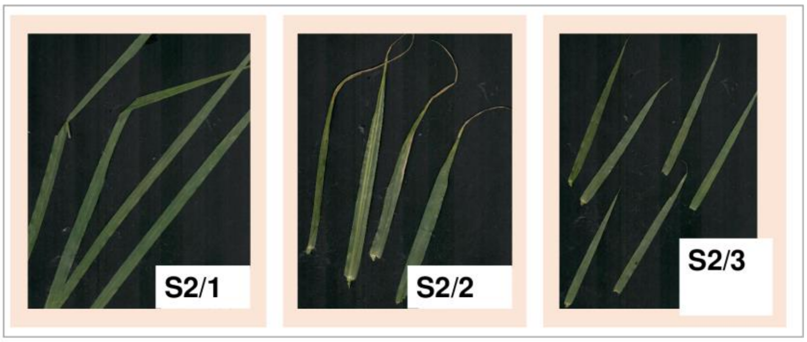

Name of the sampled plant	sRNA HTS library		Symptoms	WSMV	BYSMV	BVG	ApGIV	LDV1	n of infecting viruses
Setaria viridis	6_ECGSV_BA	S2/1	Mosaic symptoms	1					1
		S2/2	M, Ldef, TN, Chl	1					1
		S2/3	Mosaic symptoms	1					1

(i)

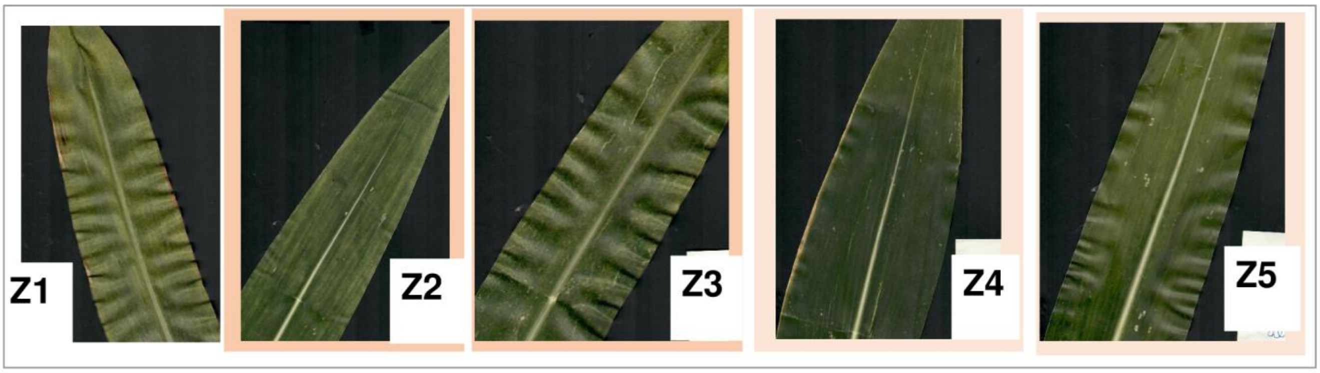

Name of the sampled plant	sRNA HTS library		Symptoms	WSMV	BYSMV	BVG	ApGIV	LDV1	n of infecting viruses
Zea mays	7_Ma_BA	Z1	Chl, M, Ldef						0
		Z2	M, Stu, Chl				1	1	2
		Z3	Chl, M, Stu				1	1	2
		Z4	Stunning					1	1
		Z5	Chl, Stu					1	1

Figure S2. Photos of the sampled individuals, indicating their registration number. The intensity of the background colour indicates the virus infection status. The deeper the colour the more number of viruses were found in the plant. Virus infection data are also summarized as a small table, indicating the incidence of the viral infection. (a) *Panicum miliaceum* at US, (b) *Echinocloa crus-galli* at US, (c) *Setaria viridis* at US, (d) *Cynodon dactylon* at US, (e) *Sorghum halepense* at U, (f) *Panicum miliaceum* at BA, (g) *Echinocloa crus-galli* at BA, (h) *Setaria viridis* at BA, (i) *Zea mays* at BA.

Abbreviations used for viral symptoms are: Ldef – leaf deformation; M – mosaic; MM – mild mosaic, Chl – chloro-sis; MC – mild chlorosis, Mo – mottle, P – Purple colouration; N – necrosis; TN – tip necrosis, VN – vein necrosis, Stu – stunting; TN – tip necrosis.
